# Supplementary material for: Splice-Junction-Based Mapping of Alternative Isoforms in the Human Proteome
Source: Cell Rep. Author manuscript; Available in PMC 2020 Jan 15. (PMC6961840; doi:10.1016/j.celrep.2019.11.026)
Supplement: 2 [file NIHMS1546469-supplement-2.zip › DF1/PXD010154/Spleen/Spleen_5_PPP1R12A_ISEMEEELKR.pdf]

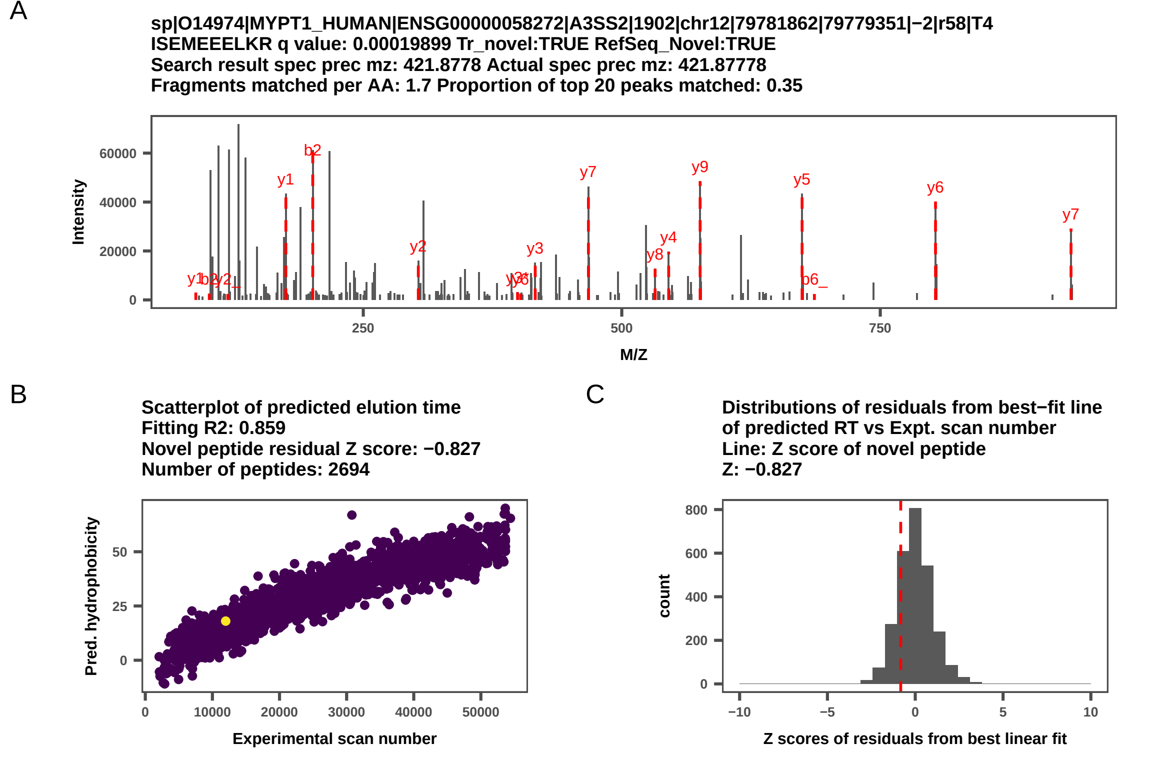
A
sp|O14974|MYPT1_HUMAN|ENSG00000058272|A3SS2|1902|chr12|79781862|79779351|−2|r58|T4
ISEMEEELKR q value: 0.00019899 Tr_novel:TRUE RefSeq_Novel:TRUE
Search result spec prec mz: 421.8778 Actual spec prec mz: 421.87778
Fragments matched per AA: 1.7 Proportion of top 20 peaks matched: 0.35
Intensity
60000
40000
20000
0
y1
b2
y2
y3
y7
y8
y4
y9
y5
b6_
y6
y7
250
500
750
M/Z
B
Scatterplot of predicted elution time
Fitting R2: 0.859
Novel peptide residual Z score: −0.827
Number of peptides: 2694
Pred. hydrophobicity
50
25
0
0
10000
20000
30000
40000
50000
Experimental scan number
C
Distributions of residuals from best−fit line
of predicted RT vs Expt. scan number
Line: Z score of novel peptide
Z: −0.827
count
800
600
400
200
0
−10
−5
0
5
10
Z scores of residuals from best linear fit
